# A qualitative study exploring the pros and cons of universal pediatric palliative care referral at diagnosis for children with cancer

**DOI:** 10.1017/S147895152610217X

**Published:** 2026-04-01

**Authors:** Leeat Granek, S.C.M. Veldhuijzen van Zanten, Dave Lysecki, Adam Rapoport, Natasha Datoo, Fyeza Hasan, Sumit Gupta, Lori Wiener, Kimberley Widger, Anthony Chan, Alisha Kassam, Karen Fergus, Emily McCullogh, Donna Johnston

**Affiliations:** 1 School of Health Policy and Management, York University, Toronto, Ontario, Canada; 2 Palliative Care, Children’s Hospital of Eastern Ontario, Toronto, Ontario, Canada; 3 Division of Palliative Medicine, Department of Pediatrics, McMaster University, Toronto, Ontario, Canada; 4 Paediatric Advanced Care Team, The Hospital for Sick Children, Toronto, Ontario, Canada; 5 Division of Haematology/Oncology, BC Children’s Hospital, Toronto, Ontario, Canada; 6 Division of Haematology/Oncology, Hospital for Sick Children, Toronto, Ontario, Canada; 7 Center for Cancer Research, National Cancer Institute, NIH, Toronto, Ontario, USA; 8 Lawrence Bloomberg Faculty of Nursing, University of Toronto, Toronto, Ontario, Canada; 9 Department of Paediatrics, Southlake Hospital, Toronto, Ontario, Canada; 10 Department of Psychology, Faculty of Health, York University, Toronto, Ontario, Canada; 11 Division of Haematology/Oncology, Children’s Hospital of Eastern Ontario, Toronto, Ontario, Canada

**Keywords:** Palliative care, communication, symptom management, child, neoplasms, terminal care, qualitative research, health personnel, patient referral

## Abstract

**Background:**

Early integration of pediatric palliative care (PPC) offers significant benefits for children with cancer, yet referrals often occur late in the child’s cancer trajectory.

**Objectives:**

As part of a larger project looking at barriers and facilitators to early integration of PPC, this study explored the perspectives of healthcare providers (HCPs) on the pros and cons of a universal referral system where all children with cancer are referred to PPC at diagnosis.

**Methods:**

Using the grounded theory method, semi-structured interviews were conducted with 66 oncology and PPC providers across 4 tertiary cancer centers in Canada. Interviews were coded line-by-line to explore patterns and themes across the dataset.

**Results:**

Three key benefits emerged that included: reducing stigma and normalizing PPC as standard care, fostering early relationship building with patients and families, and minimizing HCP subjectivity in making PPC referrals. Cons included the idea that universal referral was a poor use of resources, particularly for children with curable cancers, and that this system lacked usefulness for patients and families.

**Significance of results:**

Universal referral can promote equitable, timely, and family-centered integration of PPC in pediatric oncology. However, these types of referral systems face substantial challenges, particularly around resources. There was also wide variation of opinions and acceptability of universal referral among providers. The adoption of standardized or tiered referral criteria, guided by disease risk, prognosis, or symptom burden, may represent a practical middle ground. Future work should evaluate the impact of such criteria-based referral models on patient and family outcomes, provider experiences, and healthcare resource use.

## Introduction

Pediatric palliative care (PPC) is a holistic approach to care that aims to alleviate physical, psychological, and spiritual distress caused by serious illness and its treatment (Field and Behrman 2003). Historically, PPC was seen as synonymous with end of life (EOL) (Sisk et al. 2020); however, research has repeatedly found that children with cancer can benefit from PPC integration early in their cancer trajectory (Weaver et al. 2015; Holder et al. 2024). For example, a recent systematic review of 32 papers that included more than 15,000 children and 342 parents in total concluded that children with cancer who had received PPC reported improved symptom burden, better pain control, increased quality of life with fewer invasive procedures, increased completion of advance care planning conversations and resuscitation status documentation, and fewer deaths in hospital in the intensive care unit (Kaye et al. 2021). Despite the clear benefits of PPC services, early integration of PPC is not commonly practiced, and many children who can benefit from PPC either never obtain these services or receive them late in their cancer trajectory (Holder et al. 2024).

## Canadian context

In a large national project, our team set out to explore barriers and facilitators to the early integration of PPC for children with cancer at 4 pediatric hospitals across Canada. At one of these sites, *The Children’s Hospital of Eastern Ontario* (CHEO), referral to PPC is universal following a diagnosis of cancer for all patients with the exceptions of those for whom management is exclusively surgical. The initial consult occurs shortly following diagnosis and consists of an explanation of their program and the services they provide. For patients with high-risk disease or high symptom burden requiring management (e.g., high-risk neuroblastoma, Acute Myeloid Leukemia (AML), etc.), palliative care follows these patients regularly regardless of whether they are admitted on the inpatient unit or are in the outpatient clinic. For patients with lower risk disease (e.g., standard risk Acute Lymphoblastic Leukemia. (ALL), low-risk Wilms’ tumor, etc.), only an initial consultation is completed unless there are significant pain and symptom management issues in which case the palliative care team continues to see the child regularly until symptoms are resolved. For the remainder of patients, palliative care remains involved and engages as required based on symptoms and disease trajectory. The other 3 sites in the study do not have a universal referral process at diagnosis. The purpose of this sub-study was to explore the perceived benefits and disadvantages of the universal referral system where children with cancer are referred to PPC at diagnosis through the lens of healthcare providers (HCPs).

## Methods

### Participants and procedure

This study used the grounded theory method (Charmaz 2006) and was part of a larger project exploring the barriers and facilitators to the early integration of PPC for children with cancer (Granek et al. 2026). As part of this project, we published a paper that presented a systems theory model to understand the process of PPC referral for children with cancer (Granek et al. 2026). Prior to launching the study, Research Ethics Boards Approvals were obtained at 4 participating sites in November 2023 (University of British Columbia; REB: H23-01211l; Hospital for Sick Children, CHEO, and McMaster University were approved through Clinical Trials Ontario Centralized REB, Project Id: 4337). Participants were recruited from 4 major Canadian pediatric centers that have specialized PPC teams and treat a significant proportion of Canadian children with cancer. Inclusion criteria were being a HCP with at least 1 year’s experience, who primarily treats pediatric patients with cancer, and/or provides PPC. Eligible professions included physicians, nurses, social workers, and other interprofessional health team members. Participants were recruited by study co-investigators who emailed staff that met inclusion criteria. Signed consent was obtained. A semi-structured interview guide was used. Interviews took place between February 2024 and June 2024 and were conducted by the research associate, a female postdoctoral fellow with extensive experience conducting qualitative research. The interviewer was not a HCP and did not know any of the participants or have any prior assumptions about PPC prior to the launch of the study. The interview guide was developed by the research team and questions focused on barriers and facilitators to early PPC referral for children with cancer. As part of this flexible interview guide, participants were asked to reflect on whether they had any universal systems for referral to PPC in place and nearly half of the participants were directly asked to comment about the universal referral system at CHEO where all children with cancer receive a PPC consult at diagnosis. Interviews were conducted using videoconferencing technology, audio-recorded and lasted between 45 and 75 minutes. Interviews were then transcribed and de-identified for coding.

### Data analysis

The RA conducted the data analysis in close collaboration with the principal investigator (PI). The RA and the PI independently coded the first 5 transcripts and met to discuss the developing coding scheme to work out any differences in interpretation of the data and the labelling of the codes. Subsequently, the RA coded the remainder of the transcripts with weekly consultation with the PI. Data collection and analysis occurred concurrently, with line-by-line coding of transcripts guiding the process (Charmaz 2006). Codes were developed iteratively and no preconceived codes or categories were used. Throughout the study, the PI and the RA wrote memos about emerging ideas from the analysis and the team met regularly to discuss emerging findings, ensure consistency, and refine the coding scheme. NVivo 12 software was used to code, store, and organize the data. When the coding was complete, the PI pulled out all the themes and sub-themes related to universal palliative care referral for this study. Data collection stopped when data saturation was reached and no new codes were created.

## Results

Participant demographics are presented in Table 1. Of the 66 participants, 25% work at CHEO (17/66). Forty seven percent (31/66) of participants were asked directly about their thoughts on CHEO’s universal referral system at diagnosis for all children with cancer. Another 24% (16/66) of participants brought up automatic referrals and/or the CHEO system spontaneously without being asked about it directly, and 29% (19/66) were not asked about universal referrals or the CHEO system and it did not come up in their interviews.

**Table 1.** Participant demographics (*N* = 66)

Characteristic	
Gender % (*N*)	
Male	22.7 (15)
Female	77.3 (51)
Age mean (SD)	41.9 (10.2)
Role % (*N*)	
Oncologist	25.8 (17)
Oncology nurse	19.7 (13)
PPC physician	18.2 (12)
PPC nurse	10.6 (7)
Oncology pharmacist	6.1 (4)
Oncology social worker	4.5 (3)
Oncology child life specialist	3.0 (2)
Oncology dietician	3.0 (2)
PPC child life specialist	3.0 (2)
Oncology physiotherapist	1.5 (1)
PPC social worker	1.5 (1)
PPC recreational therapist	1.5 (1)
PPC grief support worker	1.5 (1)
Years in role % (*N*)	
<1–10	60.6 (40)
11–20	24.2 (16)
21–29	7.6 (5)
30 or more	7.6 (5)
No. of patients seen per week % (*N*)	
1–10	12.1 (8)
11–20	30.3 (20)
21–30	15.2 (10)
41–50	16.7 (11)
>50	18.2 (12)
Does not know	1.5 (1)

### Benefits of automatic PPC referral

Participants reported 3 main benefits to the universal referral system at diagnosis included: (1) *Reducing Stigma and Normalizing PPC as a Standard of Care*; (2) *Building Relationships with Patients and Families*; and (3) *Reducing HCP Subjectivity in Making Referrals to PPC.*

**Reducing Stigma/Normalizing PPC as Standard of Care**: Participants noted that a universal referral process to PPC reduced the stigma around PPC being primarily about “end of life” or a service that was called in when there was “nothing else to do” for a patient. It normalized PPC as a standard of care for all oncology patients and eliminated parental shock when PPC was consulted late in a child’s diagnosis. One palliative care physician noted: “I think [universal referral] made introducing and normalizing that we’re part of the care team easier, because it’s everybody … just normalizes it. I think it diminishes the stigma.” (P. 22)

**Building Relationships with Patient/Family**: Participants reported that the universal referral system allowed the PPC team to build strong relationships and familiarity with the patient and the family long before they would need their services. Illustrating this point, 1 oncology nurse reflected:

At the beginning, when we weren’t automatically referring our patients, I think the palliative care team felt that they would get involved too late with the patient. It would really be at the end, when the patient is in crisis. Then they didn’t have a rapport with the families. So that is one of the reasons why they do want to get involved, the sooner the better. It’s to get that relationship before you get to the end. (P. 31)

This relationship building was perceived to increase the patient and family’s receptivity to accepting PPC services, particularly during crisis situations (i.e., relapse, complications), but especially when the child was nearing the EOL and needed increased support by the PPC team. One PPC physician explained:

Often, we get introduced at a time when it’s clear that we have to be introduced. That’s much harder to build a relationship with a family when you’re being introduced, because they relapsed, because there’s a metastatic scan, like a bad scan, then they don’t want to meet us because then they know that it’s truly symbolic of their child’s impending death. I would love for it to be more integrated as standard of care for all cancers. (P. 10)

**Reducing HCP Subjectivity in Making Referrals to PPC**: Lastly, participants explained that the universal referral system reduced HCP subjectivity as to when a family should be referred to PPC. In this context, participants reported that referral to PPC was often the choice of individual oncologists and that there was wide variation in the way they referred. The universal referral system took out all subjectivity from this decision-making and eliminated the “guesswork” as to when was the right time to refer to PPC. Reflecting on this issue, 1 PPC physician explained:

I would say some of the oncologists are very uncomfortable with palliative care. But it’s much easier to say, “Hey, we refer everybody to this team, you know, you’ll meet them.” It’s actually made it much easier because some of the oncologists who have the more complicated patients were the oncologists who were more uncomfortable introducing our team. (P.44)

### Cons of the universal referral system at diagnosis

Participants named 2 primary cons to the universal referral system that included: (1) *Resource and Volume Concerns* and (2) *System Lacking Usefulness*.

**Resources and Volume**: Participants noted that in large volume hospitals where hundreds of patients with newly diagnosed cancer are treated each year, referring each child to PPC would not be feasible in terms of cost and work demands on the PPC team, and would be a poor use of resources. On this, 1 social worker remarked: “I don’t know that every single newly diagnosed cancer patient [makes sense] – I’m not sure that’s a good use of resources to send everyone to PPC. We have hundreds of kids a year, hundreds. They certainly wouldn’t have the capacity to meet every single new cancer diagnosis.” (p. 59)

Another oncologist who also worked in administration similarly noted that this system did not seem feasible within already constrained health systems. They explained:

I’m wearing my administrative hat, I’m not sure that’s feasible. I know they meet every patient who gets a solid organ transplant. … I also think there needs to be a triage because to meet someone and then not use [those services] for four years, I’m not sure what the value out of that is. I think in a resource unlimited world, it might be okay. (P. 52)

**System Lacking in Usefulness**: Related to the point above about resources and value of such a system, participants also questioned the usefulness of a universal PPC referral system where it was felt that many children could have their symptoms successfully treated by the oncology team and were likely to have a good outcome to their disease. On this, 1 oncologist remarked:

I don’t think that every child with cancer needs a palliative care referral. I realized that they could do more symptom management for those patients and maybe have a little more time to think about that than we do. But for the standard chemotherapy-related symptoms, I think that we do a pretty good job managing that. … I am worried that PPC resources would be spent on a large proportion of patients who have let’s say, standard risk leukemia, or low risk tumors, or patients who are almost certainly going to be cured, who definitely have a journey to go through, but who need that expertise a little bit less than some of our patients who needed quite a bit. I’d be worried about a resource drain. Although I would like the idea of an automatic referral, I don’t want it for every cancer patient. I think there should be another guideline of who that can be for. (p. 43)

Another point about the usefulness of this system was made by another oncologist who felt that the universal “meet and greet” system with the PPC team did not necessarily translate into useful services for the families but came at high resource cost for the team. They remarked:

I don’t think that’s useful at all. I think what happens is, is either they come in and they say “Hi” and they’re not providing anything. I just don’t know how it’s helpful, especially in our model here where the primary palliative care team is not the team here, it’s going to be the home team. So, meeting the PPC team near a diagnosis to have them come in and say, “Hi,” every two weeks? I haven’t found that useful. I know the PPC team disagrees with that, but we just haven’t found it useful when this happens. And I think a lot of centers would agree with that. (P. 58)

## Discussion

### Main findings

These findings highlight the potential benefits and limitations of a universal referral system to PPC from the perspective of HCPs working in oncology and palliative care settings in children’s hospitals across Canada. Participants emphasized how this universal process helped reduce stigma by reframing PPC as a routine part of oncology care rather than a service solely associated with EOL. This normalization appears to improve acceptance and understanding of PPC among families and patients and changed the perception that there is only a role for PPC involvement when someone is dying. Additionally, early and consistent PPC involvement facilitated by universal referral was seen as key to fostering relationships between the care team and families and was viewed as critical in enhancing trust and communication, particularly during moments of crisis or disease progression.

### What this study adds

A universal referral system was also seen as a mechanism to reduce variation and subjectivity in referral practices, helping to standardize care across providers. This finding suggests that this universal process can address potential biases or inconsistencies in clinical judgment, promoting equitable access to PPC. This last point is especially critical as research has consistently documented that barriers to refer to PPC may be attributed to the medical community’s general focus on curative treatment and the belief that PPC should only be offered at EOL (Hawley 2017; Sarradon-Eck et al. 2019; Laronne et al. 2021; Alcalde and Zimmermann 2022; Holder et al. 2024). It is well understood that the transition from a focus of cure to supportive care can bring up feelings of failure, guilt, grief, and sadness in HCPs (Granek et al. 2012) resulting in the first introduction to PPC often occurring when all curative treatment options are exhausted. The themes that emerged from our data suggest that universal PPC referral systems may improve both the timeliness and quality of care for pediatric oncology patients, while also supporting HCPs in delivering more consistent and patient-centered care.

While universal referral has some clear benefits, our study also highlighted that implementing such a system may not be feasible in heavy volume centers where resources are already limited. While it can be argued that PPC resources can be increased if it became more integrated as a standard of care, some participants still felt that universal consults with the PPC team did not provide enough value for the costs and that the PPC needs of children with lower risk disease could be successfully met by the primary multidisciplinary oncology team. This difference of opinion reflects the dominant tensions within pediatric oncology teams when it comes to PPC integration and is also reflected in our finding that participants felt that a PPC referral was often a subjective decision made by the treating oncologist.

### Clinical implications

Given the clear benefits of the universal referral system for children with cancer, pediatric cancer departments might consider the feasibility of such a system for their own centers where PPC referrals may be occurring late in the child’s disease course. Where a universal referral system is not feasible, centers might consider hybrid or tiered referral models based on disease risk, treatment intensity, and symptom burden. Several published studies support the feasibility and value of establishing standardized referral criteria systems for specialist PPC and have identified a need to standardize these criteria for HCPs (Hui et al. 2016; Cuviello et al. 2021b; Bernier Carney et al. 2023). For example, Cuviello and colleagues found that 85% of pediatric oncology providers endorsed a screening tool to standardize PPC referral, with common triggers including symptom burden and poor prognosis (Cuviello et al. 2021a).

These findings suggest that implementing standardized referral criteria may help normalize PPC integration, reduce variability in practice, and improve early access for children with serious cancer. In the context of our study, this supports the recommendation that oncology programs consider not only universal referral systems but also clearly defined criteria for PPC referral at diagnosis or early in the trajectory, tailored to children with higher risk or symptom burden.

*Limitations*: This study used a semi-structured interview guide where all participants were asked a core set of questions, but as is standard with qualitative research, also allowed for flexibility in the interview guide for participants to bring up topics that were important to them. As such, while the majority of participants in this study discussed the CHEO system or an automatic referral system for PPC, some participants were not probed about this and did not bring it up in their interviews, and therefore, the findings may not reflect the views of the 29% of participants who did not comment on automatic referrals. Further research might use quantitative methods to systematically assess the acceptability of a universal referral system among pediatric oncology teams. Secondly, we recruited at 4 large tertiary hospitals in Canada. Differences in institutional structures, staffing ratios, and PPC program maturity across sites may have influenced participants’ views, limiting the comparability of perceptions about feasibility.

## Strengths and conclusion

This study contributes to the growing evidence supporting the early and systematic integration of PPC for children with cancer. HCPs across Canadian pediatric centers recognized that universal referral systems at diagnosis can normalize PPC as a standard part of oncology care, reduce stigma, and foster early relationship-building between families and care teams. These processes may also minimize provider subjectivity in referral decisions and promote more equitable access to supportive services. However, the feasibility of universal referral remains limited by resource constraints in high-volume centers. As such, the adoption of standardized or tiered referral criteria, guided by disease risk, prognosis, or symptom burden, may represent a practical and evidence-informed middle ground. Future work should evaluate the impact of such criteria-based referral models on patient and family outcomes, provider experiences, and healthcare resource use. Developing context-appropriate referral frameworks in Canada will be a critical next step toward ensuring that all children with serious illness receive timely and comprehensive palliative care.
